# Major HGF-mediated regenerative pathways are similarly affected in human and canine cirrhosis

**DOI:** 10.1186/1476-5926-6-8

**Published:** 2007-07-31

**Authors:** Bart Spee, Brigitte Arends, Ted SGAM van den Ingh, Tania Roskams, Jan Rothuizen, Louis C Penning

**Affiliations:** 1 Department of Clinical Sciences of Companion Animals, Faculty of Veterinary Medicine, Utrecht University, The Netherlands; 2 Department of Morphology and Molecular Pathology, University Hospitals Leuven, Belgium

## Abstract

**Background:**

The availability of non-rodent animal models for human cirrhosis is limited. We investigated whether privately-owned dogs (*Canis familiaris*) are potential model animals for liver disease focusing on regenerative pathways. Several forms of canine hepatitis were examined: Acute Hepatitis (AH), Chronic Hepatitis (CH), Lobular Dissecting Hepatitis (LDH, a specific form of micronodulair cirrhosis), and Cirrhosis (CIRR). Canine cirrhotic samples were compared to human liver samples from cirrhotic stages of alcoholic liver disease (hALC) and chronic hepatitis C infection (hHC).

**Results:**

Canine specific mRNA expression of the regenerative hepatocyte growth factor (HGF) signaling pathway and relevant down-stream pathways were measured by semi-quantitative PCR and Western blot (STAT3, PKB, ERK1/2, and p38-MAPK). In all canine groups, levels of c-MET mRNA (proto-oncogenic receptor for HGF) were significantly decreased (p < 0.05). Surprisingly, ERK1/2 and p38-MAPK were increased in CH and LDH. In the human liver samples Western blotting indicated a high homology of down-stream pathways between different etiologies (hALC and hHC). Similarly activated pathways were found in CIRR, hALC, and hHC.

**Conclusion:**

In canine hepatitis and cirrhosis the major regenerative downstream pathways were activated. Signaling pathways are similarly activated in human cirrhotic liver samples, irrespective of the differences in etiology in the human samples (alcohol abuse and HCV-infection). Therefore, canine hepatitis and cirrhosis could be an important clinical model to evaluate novel interventions prior to human clinical trials.

## Background

Chronic hepatitis (CH) and end-stage cirrhosis (CIRR) are an increasing medical problem, affecting over 5% of the world population [1,2]. The best-studied animal model for these liver diseases is tetracarbon-induced fibrotic liver diseases inflicted in rats [3]. Many more models have been devised to mimic liver diseases in man; the time-course of the development however, is not always comparable to the human situation [4-8]. Furthermore, the variability in the affected human population regarding, sex, age, social factors, eating- and drinking behavior, body weight etc. is not fully covered in standardized laboratory conditions.

**Figure 1:**
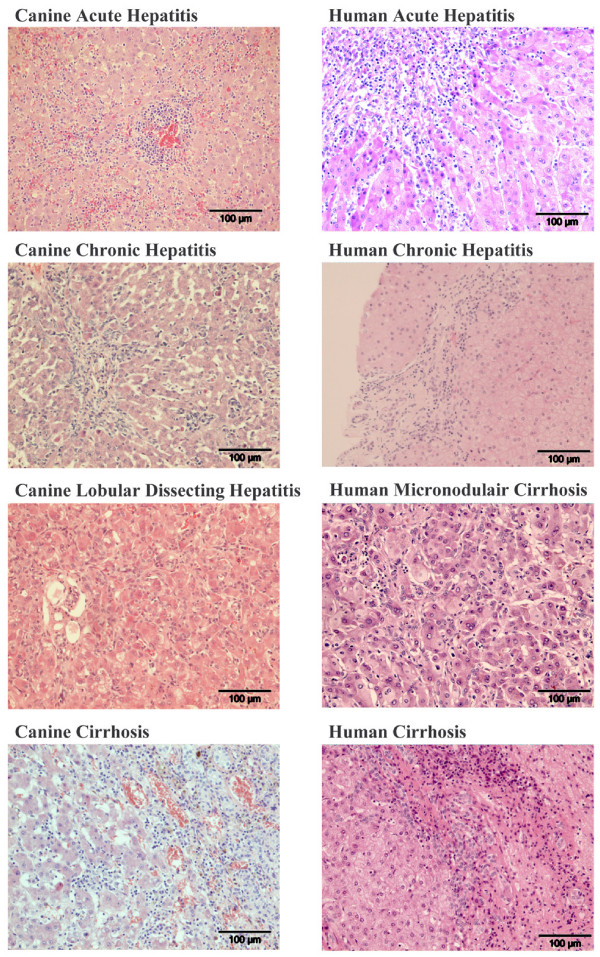
**Examples of histological samples (HE) from canine and human liver diseases**. CANINE ACUTE HEPATITIS: Marked infiltration of mononuclear and granulocytic inflammatory cells and focal necrosis. CANINE CHRONIC HEPATITIS: Small fibrous septum with interface hepatitis as well as lobular hepatitis; several apoptotic bodies. CANINE LOBULAR DISSECTING HEPATITIS: Lobular dissection by fibroblasts and ductular proliferation and a slight inflammatory infiltrate. CANINE CIRRHOSIS: Broad fibrous septum with ductular proliferation and a marked inflammatory infiltrate with interface hepatitis. HUMAN ACUTE HEPATITIS: Marked infiltration of mononuclear and some granulocytic inflammatory cells and some apoptotic bodies. HUMAN CHRONIC HEPATITIS: Fibrous septum with some ductular proliferation and slight interface hepatitis. HUMAN MICRONODULAR CIRRHOSIS: Dissection of the parenchyma by small fibrous septa, slight ductular proliferation and inflammation; hepatocytic ballooning, Mallory bodies, and apoptosis. HUMAN CIRRHOSIS: Broad fibrous septum with ductular proliferation and a marked inflammatory infiltrate with interface hepatitis.

Dogs have liver diseases which are clinically highly comparable with the human counterparts and both species have a high resemblance at the genetic level [9,10]. In contrast to rodent models, hepatitis in these dogs is not deliberately induced. Previous studies already showed a high resemblance between man and dogs in the formation of fibrosis during liver diseases [11]. Furthermore dogs share many years in close proximity of humans exposing them to the same environmental and biological stresses. Recently, detailed clinical and histological diagnostic standards have been published for all liver diseases of dogs [12]. Corroborating the histological similarities between human and dogs, examples are provided in Figure 1. Taken together, these similarities suggest the dog as a model animal for liver diseases in man [13].

Liver regeneration is a complex interplay of different factors [14]. One of the main growth factors identified in liver regeneration is Hepatocyte Growth Factor (HGF). HGF activates the proto-oncogenic receptor tyrosine kinase c-MET and subsequent down-stream pathways, including the anti-apoptotic protein kinase-B (PKB/Akt) cascade, the proliferative MAP-kinase pathway (ERK1/2 and p38MAPK), and the STAT3 signaling (signal transducers and activators of transcription) [15-17]. At present, a comparison between these regeneration signal transduction pathways in human and canine livers is missing, hampering the application of dogs as (pre-) clinical model animals for human medicine.

We have analyzed HGF-mediated regeneration signaling in canine samples from dogs with Acute Hepatitis (AH), Chronic Hepatitis (CH), and Lobular Dissecting Hepatitis (LDH, a specific form of micronodulair cirrhosis similar to neonatal hepatitis in human hepatology) [18,19], and CIRR. The cirrhotic samples were compared to two human cirrhotic diseases with different etiologies; HCV-induced (hHC) and alcohol-induced (hALC). This study will elucidate the potential of (non-experimental) dogs to bridge between toxin-induced rodent models and human clinical situation.

## Results

### HGF/c-MET signaling pathways involved in liver regeneration in dogs with AH, CH, CIRR, and LDH

**Figure 2:**
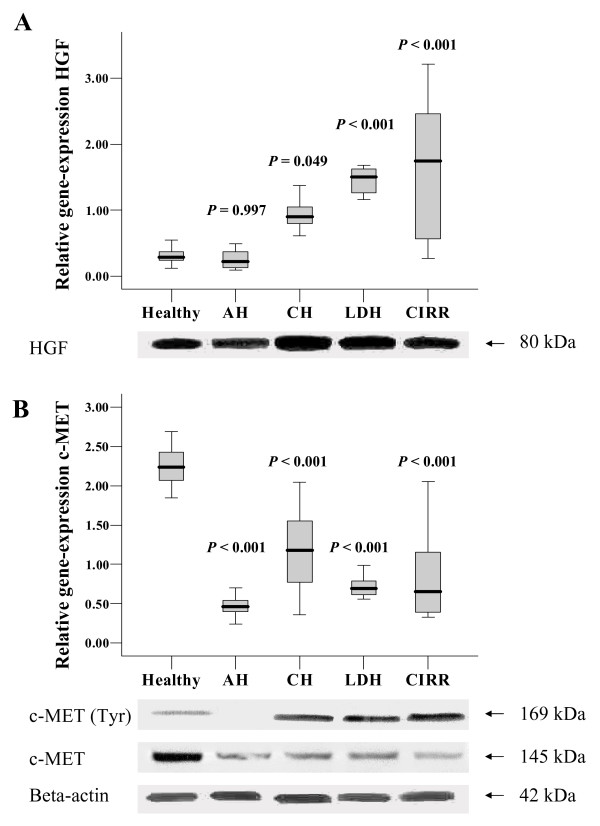
**Quantitative Real-Time PCR of genes involved in regeneration and growth**. Expression analysis of acute hepatitis (AH), chronic hepatitis (CH), lobular dissecting hepatitis (LDH), cirrhotic samples (CIRR). HGF expression and detection of the 80 kDa HGF protein is shown in (A). c-MET gene expression and detection of c-MET including phosphorylated (Tyr) form is shown in (B). Relative gene expression data represent mean + SE. A Kolmogorov-Smirnov test was performed to establish a normal distribution and a Levenes test for the homogeneity of variances. All samples included in this study were normally distributed. The statistical significance of differences between diseased and control animals was determined by using the two-sided Dunnetts post-test analysis. A *P*-value < 0.05 was considered statistically significant. Analysis was performed using SPSS software (SPSS Benelux BV, Gorinchem, the Netherlands).

Statistically significant differences (ANOVA) were identified in the expression of mRNA encoding HGF (*P *< .001) and c-MET (*P *< .001). Towards healthy control samples HGF mRNA levels were significantly induced in CH, LDH, and CIRR, three-, five-, and five-fold, respectively (Figure 2A). In AH, HGF mRNA levels remained unchanged toward healthy control. Western blot analysis on HGF protein showed the presence of a HGF band (80 kDa) in all canine samples. There is a clear quantitative correlation between HGF protein and mRNA levels, as both HGF mRNA and protein levels are increased in CH, LDH, and CIRR. The c-MET mRNA levels in all groups were significantly decreased towards control, with a maximum four-fold reduction in AH (Figure 2B). Total c-MET protein levels are similarly reduced as the mRNA levels. Analysis on phosphorylated c-MET showed an immuno-reactive band in CH, LDH, and CIRR, which was almost absent in AH.

### Western blot analysis on STAT3, PKB/Akt, ERK1/2, and p38MAPK in canine liver homogenates

**Figure 3:**
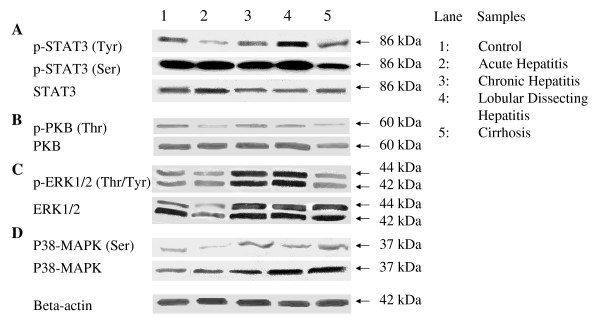
**Western blot analysis of canine liver homogenates of several diseases (n = 6)**. Western blot analysis of pooled canine liver homogenates in acute hepatitis (AH), chronic hepatitis (CH), lobular dissecting hepatitis (LDH), cirrhotic samples (CIRR). Detection of the 86 kDa STAT3 protein (A), detection of the 60 kDa PKB protein (B), detection of the 44/42 kDa ERK 1/2 protein shown in (C), and detection of the p38-MAPK protein shown in (D). Beta-actin was used as a loading control.

In Figure 3 important downstream signaling proteins of HGF/c-MET (STAT3, PKB/Akt, ERK1/2, and p38-MAPK) are depicted. The total STAT3 was detected as an immuno-reactive 86 kDa band in all hepatic diseases, with slightly lower expression in CH, LDH, and CIRR. Tyrosine (Tyr)-phosphorylated STAT3 was strongly reduced in AH and was increased in LDH. Healthy controls, CH, and CIRR groups had comparable levels of Tyr-phosphorylated STAT3. Serine (Ser)-phosphorylated STAT3 was slightly decreased in CIRR. PKB/Akt plays a pivotal role during regeneration and growth. Total PKB/Akt was detected in all hepatic diseases as a single 60 kDa protein. Threonine (Thr)-phosphorylated PKB/Akt was less present in AH and CIRR, whereas CH and LDH showed no apparent quantitative differences toward healthy controls. Analysis of total ERK1/2 (42/44 kDa), a principle kinase in growth factor signaling, showed a slight reduction in AH, and moderate increases in CH, LDH, and CIRR. The phosphorylated form threonine/tyrosine (Thr/Tyr)-phosphorylated ERK1/2 was strongly increased in CH and LDH. A similar effect can be seen in p38-MAPK, an increased expression of total p38-MAPK in CH, LDH, and CIRR and decreased levels in AH. The threonine/tyrosine (Thr/Tyr)-phosphorylated p38-MAPK form is increased in CH, LDH, and CIRR.

### Western blot analysis on human cirrhotic explant samples after alcohol abuse (hALC) and after hepatitis C virus infection (hHC)

**Figure 4:**
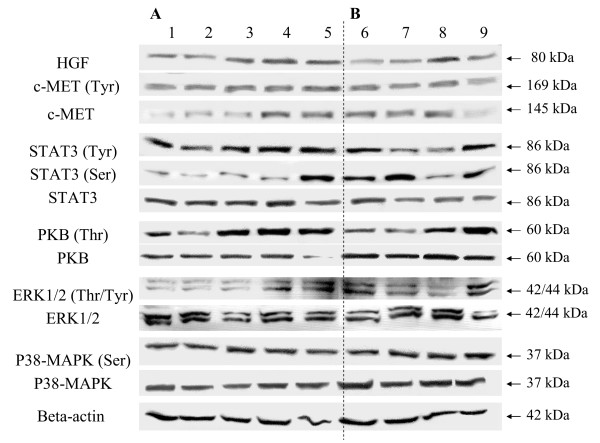
**Western blot analysis of human liver homogenates of several diseases (n = 6)**. Samples 1 to 5 represent individual alcoholic cirrhosis (hALC) shown in (A), samples 6 to 9 represent individual hepatitis C induced cirrhotic samples (hHC) shown in (B). Beta-actin was used as a loading control.

Western blot analysis on HGF in hALC and hHC samples showed a detectable 80 kDa HGF in all samples with minor quantitative differences (Figure 4). The 145 kDa c-MET was also detected in all samples. The phosphorylated form of c-MET (Tyr) was detected in all samples. Total PKB/Akt was detected in all samples. The phosphorylated PKB (Thr) indicated moderate to high levels in most cirrhotic samples. Total STAT3 was detectable in all individual samples. STAT3 (Tyr) was detected in all groups although two samples in the hHC group were less phosphorylated. In general the serine phosphorylated STAT3 is lower in the hALC group compared to the hHC group. Total ERK1/2 was detected in all samples with no apparent quantitative differences. Phosphorylated ERK1/2 was detectable in all samples with different degrees of phosphorylation. Both total p38MAPK as well as the serine phosphorylated form was detected in all samples with no apparent quantitative differences.

## Discussion

To investigate if hepatic regenerative signal transduction pathways are similarly affected in liver diseases between man and dogs, the expression of HGF and c-MET was measured. Furthermore, Western blot analysis was used to show (de)activation of important signaling pathways of liver regeneration. This provided insight into major regeneration pathways in AH, CH, LDH, CIRR. A comparison between canine cirrhosis and human cirrhotic samples with different etiologies provided additional information on the suitability of the canine-model.

The increased mRNA levels of HGF and the decreased levels of c-MET mRNA in fibrotic canine diseases (CH, LDH, and CIRR), were in line with publications from human samples [20]. Together this suggests that HGF-mediated regeneration in human cirrhosis is similarly affected in canine CH, LDH, and CIRR. The reduced c-MET protein levels indicate a utilization of c-MET which is degraded through endocytosis after phosphorylation. Intracellular degradation is dependent on the interaction with Cbl ubiquitin ligases [21]. Semi-quantitative hepatocyte proliferation studies on canine samples with a Ki67 antibody indicate a moderate hepatocyte proliferation in CH; whereas cirrhotic samples (CIRR and LDH) as well as healthy samples are virtually negative (data not shown). Similar results have been described in human chronic and cirrhotic liver samples, irrespective of the etiology [22,23]. This indicates the ability of hepatocytes to proliferate after c-MET activation in more chronic stages while cirrhotic samples do not complete the cell-cycle of which the cause remains to be elucidated.

To further substantiate the molecular comparison between human and canine fibrotic livers the activation status of HGF/c-MET downstream signaling components involved in regeneration was analyzed. HCV cirrhotic samples had high levels of Ser-phosphorylated STAT3 compared to alcohol induced cirrhotic samples. This HCV-induced up regulation of STAT3 phosphorylation was shown before in man and rodent models *in vivo *as well as *in vitro *studies on cell-lines [24,25]. In other studies the levels of STAT phosphorylation indicated phosphorylated STAT3 protein in HCV affected cirrhotic livers compared to primary biliary cirrhotic samples and healthy tissue [26]. In the canine samples including the healthy control biopsies STAT3-Ser phosphorylation was strongly present. The levels of STAT3-Tyr phosphorylation were the lowest in AH and strongest in LDH. As the amount of STAT3-Tyr phosphorylation indicates the DNA binding capacity of the protein, the pathways seems to be activated in fibrotic diseases and not in acute hepatitis.

Next to STAT3 phosphorylation, HCV induces ERK1/2 phosphorylation [27]. In the canine samples, an up regulation of the levels of phosphorylated ERK1/2 was observed in CH and LDH. Finally, the increase in p38MAPK phosphorylation as observed in CH, LDH, and CIRR has been described in fibrotic tissues in man [28]. In general, HGF/c-MET downstream signaling in CH, LDH, and CIRR is to a high degree comparable with the molecular data obtained from human clinical samples.

Surgical animal models for liver regeneration, such as partial hepatectomy (PH), represent an over-simplification by the absence of inflammation or overperfusion; furthermore, all hepatocytes are stimulated by PH to enter the G1 phase simultaneously. Toxic models induced by dimethylnitrosamine, CCl_4_, acetominophen, or thioacetamide can represent chronic as well as acute/fulminant hepatitis [29-31]. Toxic models are better clinical models as hepatotoxins can be used to selectively induce centrolobular and periportal necrotic lesions and thus mimic clinical liver diseases. However, toxin-induced models do not represent the full range of changes seen in human liver diseases [32].

Comparison between canine and human diseases was obtained by using cirrhotic human samples derived from alcohol abuse or hepatitis C infection, two of the most common causes of hepatitis in the Western world [33,34]. As in humans, chronic hepatitis in dogs is associated with progressive fibrosis, reduction in liver size and regeneration, and finally disruption of the liver architecture (cirrhosis), which may cause portal hypertension, ascites, and portosystemic encephalopathy [35]. Although histologically highly comparable to their human counterparts, the etiology of canine hepatitis is largely unknown [36]. However, human samples (hALC and hHC) showed the same degree of activation in the signal transduction pathways, irrespective of the different underlying etiology. Therefore, despite unknown etiology in dogs the underlying mechanisms are similarly activated.

## Conclusion

This study is the first to measure expression profiles of crucial pathways of liver regeneration in canine liver diseases in comparison with man. Previously, a high similarity of affected fibrotic pathways between human- and canine-liver diseases was found [11]. Combining these measurements on fibrotic- and regenerative-signaling pathways, privately owned dogs may help to fill in the gap between toxin-induced rodent models and human diseases. Furthermore, this study provides the basis to analyze more acute forms of hepatitis such as (sub)acute hepatitis in dog. Taken together, these results indicated that CH, LDH, and CIRR are suitable spontaneous large animal models to evaluate the clinical application of therapies such as cell transplantation or the administration of growth factors.

## Methods

### Animals

All samples were obtained from privately owned canines of different breeds referred to our veterinary clinic. All procedures were approved by Utrecht University's Ethical Committee, as required under Dutch legislation. Each disease group (n = 11 dogs) was compared to age-matched healthy control dogs (n = 12), without clinical signs of hepatitis or other disease (histopathology did not reveal any abnormalities). Liver biopsies were obtained (ultrasound-guided) from all dogs under local anesthesia with a true cut 14G biopsy needle, preceded by ultrasonographic evaluation of the liver to exclude non-homogeneous hepatic changes. Two formalin-fixed biopsies were embedded in paraffin, sliced, and stained with hematoxylin and eosin-, van Gieson-, and reticulin-stain according to Gordon and Sweet. All histological examinations were performed by one experienced, certified veterinary pathologist. Two other biopsies were snap-frozen and stored at -70°C until molecular analysis.

### Human patients

All liver samples were obtained from surgical patients transplanted at the Department of Abdominal Transplantation in the University Hospital Leuven, Leuven, Belgium. The procedures were approved by Leuven University's Ethical Committee, as required under Belgian legislation. Human explant samples were collected directly after surgery and immediately snap-frozen. All patients, predominantly male, were presented with micronodular cirrhosis. The Alcoholic Cirrhosis (hALC) group contained five patients (n = 5) characterized by cirrhosis with neutrophil infiltrations, alcohol related morphological changes (hepatocyte ballooning, Mallory bodies, necrosis), and in some cases steatosis and increased iron deposition. The Hepatitis C (hHC) group contained four patients (n = 4) characterized by cirrhosis with neutrophil infiltrations, lymphoid follicles and aggregates. All cases were presented with hepatocyte decay, apoptosis/necrosis, regeneration, and fibrosis.

### Quantitative PCR

Quantitative real-time PCR (Q-PCR) was performed on HGF and c-MET. The abundance of mRNA was determined by reverse transcription followed by real-time quantitative PCR using appropriate primers (Table 1), as described previously [37]. For each experimental sample, two endogenous reference genes (GAPDH and HPRT) were included. The use of two reference genes is sufficient for reliable data [38]. Results were normalized according to the average amount of the endogenous references. The relative gene-expression of each gene-product was used as the basis for all comparisons. The results were assessed for normality and homogeneity of variances using, respectively, the Kolmogorov Smirnoff and the Levene tests. The data were normally distributed and variances complied; therefore differences between means were determined by One Way Analysis of Variance (ANOVA) followed by the (2-sided) Dunnett post-test analysis of all groups towards control (healthy) samples. A *P*-value < 0.05 indicated significant changes.

**Table 1:** Nucleotide Sequences of Dog-Specific Primers for Real-Time Quantitative PCR

**Gene**	**Primer**	**Sequence (5'-3')**	**Tm (°C)**	**Product size (bp)**	**Accession number**
GAPDH	Forward	TGT CCC CAC CCC CAA TGT ATC	58	100	AB038240
	Reversed	CTC CGA TGC CTG CTT CAC TAC CTT			
HPRT	Forward	AGC TTG CTG GTG AAA AGG AC	56	100	L77488 /
	Reversed	TTA TAG TCA AGG GCA TAT CC			L77489
HGF	Forward	AAA GGA GAT GAG AAA CGC AAA CAG	58	92	BD105535
	Reversed	GGC CTA GCA AGC TTC AGT AAT ACC			
c-MET	Forward	TGT GCT GTG AAA TCC CTG AAT AGA AATC	59	112	AB118945
	Reversed	CCA AGA GTG AGA GTA CGT TTG GAT GAC			

### Immunoblot analysis

Twenty micrograms of pooled protein extracts (n = 6 dogs per group, randomly chosen) was separated by SDS-PAGE, and transferred to a Hybond ECL nitrocellulose membrane (Amersham-Biosciences, Cleveland, OH). The procedure for immunodetection was based on an ECL-Western blot analysis system, as previously described [39]. Primary antibodies are depicted in Table 2. Densitometric analysis was performed with a Geldoc2000 system with QuantityOne 4.3.0 software (Biorad). Human samples (protein load twenty micrograms) were analyzed separately; the immunoblot analysis was the same as for the dog protein extracts.

**Table 2:** Primary Antibodies used in Western blot experiments

**Antibody**	**Product size (kDa)**	**Dilution**	**Supplier**
Goat anti-human HGF	80	1:1,000	Santa Cruz
Goat anti-human c-MET	145	1:1,000	Santa Cruz
Rabbit anti-human c-MET (Tyr1230/1234/1235)	169	1:1,000	Abcam
Rabbit anti-human/dog STAT3	86	1:2,500	BD Biosciences
Rabbit anti-human phospho-STAT3 (Ser727)	86	1:1,000	Cell Signalling
Mouse anti-human phospho-STAT3 (Tyr705)	86	1:1,000	Cell Signalling
Mouse anti-human/dog PKB	60	1:250	BD Biosciences
Rabbit anti-human phospho-PKB (Thr308)	60	1:1,000	Cell Signalling
Rabbit anti-human Erk1/2	42/44	1:1,000	Cell Signalling
Rabbit anti-human phospho-ERK1/2 (Thr202/Tyr204)	42/44	1:1,500	Cell Signalling
Rabbit anti-human p38-MAPK	38	1:500	Abcam
Rabbit anti-human phospho-p38-MAPK (Thr180/Tyr182)	38	1:1,000	Abcam
Mouse anti-human/dog Beta-actin (pan Ab-5)	42	1:2,000	Neomarkers

## Abbreviations

AH – Acute Hepatitis; CH – Chronic Hepatitis; HGF – Hepatocyte Growth Factor; LDH – Lobular Dissecting Hepatitis; CIRR – Cirrhosis; ERK1/2 – Mitogen activated protein kinase (MAPK) 1 and 3; hALC – cirrhotic stage of alcoholic liver disease; hHC – cirrhotic stage of chronic hepatitis C infection; p38-MAPK – Mitogen activated protein kinase (MAPK) 14; PH – Partial Hepatectomy; PKB – Protein Kinase B; Q-PCR – Quantitative real-time PCR; STAT3 – Signal Transducer and Activator of Transcription 3.

## Competing interests

Despite this research was partially funded by the animal health company Intervet International, the authors declare that they have no potential conflict of interests.

## Authors' contributions

BS and BA contributed equally in writing the manuscript. BS performed the Q-PCR measurements. BA performed all Western blot experiments. TI examined the histochemical samples described in this manuscript. TR provided the human liver samples and participated in the study design. JR and LP participated in the study design and coordination of the different experiments. All authors read and approved the final manuscript.
